# Why do patients with anterior shoulder instability not return to sport after surgery? A systematic review of 63 studies comprising 3545 patients

**DOI:** 10.1016/j.jseint.2023.01.001

**Published:** 2023-01-20

**Authors:** Theodore P. van Iersel, Sanne H. van Spanning, Lukas P.E. Verweij, Simone Priester-Vink, Derek F.P. van Deurzen, Michel P.J. van den Bekerom

**Affiliations:** a Shoulder and Elbow Unit, Department of Orthopedic Surgery, OLVG, Amsterdam, The Netherlands; b Amsterdam Shoulder and Elbow Centre of Expertise (ASECE), Amsterdam, The Netherlands; c Department of Human Movement Sciences, Faculty of Behavioral and Movement Sciences, Vrije Universiteit Amsterdam, Amsterdam Movement Sciences, Amsterdam, The Netherlands; d Amsterdam UMC, Department of Orthopedic Surgery, University of Amsterdam, Amsterdam Movement Sciences, Location AMC, Amsterdam, The Netherlands; e Academic Center for Evidence-Based Sports Medicine (ACES), Amsterdam, The Netherlands; f Amsterdam Collaboration on Health and Safety in Sports (ACHSS), AMC/VUmc IOC Research Center, Amsterdam, The Netherlands; g Department of Research and Epidemiology, OLVG, Amsterdam, The Netherlands

**Keywords:** Shoulder instability, Capsulolabral repair, Bony reconstruction procedure, Return to sports, Reason, Consideration

## Abstract

**Purpose:**

To review athletes’ reasons not to return to sport (RTS) after surgical treatment of anterior shoulder instability, comparing capsulolabral repair and bony reconstruction procedures. The hypothesis is that the most common reason for patients unable to RTS is not due to physical inability of the shoulder.

**Methods:**

A systematic review was performed using the PRISMA (Preferred Reporting Items for Systematic Reviews and Meta-Analysis) guidelines. PubMed, Embase/Ovid, Cochrane Database of Systematic Reviews/Wiley, Cochrane Central Register of Controlled Trials/Wiley, SPORTDiscus/Ebsco, and Web of Science/Clarivate Analytics were searched in collaboration with an information specialist up to August 11, 2022. Observational and interventional studies reporting reasons for no RTS following surgical treatment of anterior shoulder instability were included. Quality assessment of studies was conducted using the Methodological Index for Non-Randomized Studies (MINORS) criteria and Risk of Bias (RoB) assessment. Forest plots were generated to show an overview of the proportion shoulder function independent reasons for each study.

**Results:**

Sixty-three studies were included reporting on 3545 athletes, of which 2588 (73%) underwent capsulolabral repair versus 957 (27%) who underwent surgical treatment with bony reconstruction procedures. A total of 650 athletes (18%) were unable to RTS. The reason not to RTS was most frequently shoulder function independent (70%) compared to shoulder function dependent (30%) following both capsulolabral repair and bony reconstruction procedures. Most cited reasons for no RTS after capsulolabral repair were fear of reinjury (17%), personal reasons or change of priorities (11%) and retirement/discharge of military service or sports team (10%). Of these reasons, 106 (22%) were not specified other than being shoulder function dependent or shoulder function independent. Most cited reasons for no RTS after bony reconstruction procedures were fear of reinjury (12%), shoulder pain (10%), and retirement/discharge of military service or sports team (9%). Of these reasons, 74 (44%) were not specified other than being shoulder function dependent or shoulder function independent. Forest plots showed a variation from 0% to 100% shoulder independent reasons for both capsulolabral repair and bony reconstruction procedures.

**Conclusion:**

The majority of athletes who did not RTS following surgical treatment for anterior shoulder instability did so due to shoulder function independent reasons, such as fear of reinjury. However, there was a high variety between studies and many reasons were unspecified, warranting unified definitions for reasons of patients that do not RTS.

Shoulder instability is highly frequent among contact/collision and overhead athletes.^13^^,^^27^^,^^91^ It can result in time-loss (eg, missed games) and high socioeconomical and hospital costs.^23^^,^^87^ Furthermore, shoulder dislocations are the most occurring dislocations of the human body, for which surgical treatment is often indicated. It is widely used after failed nonoperative treatment.^2^^,^^6^^,^^78^ Even though return to sport (RTS) rates are high in current literature, up to 19% of patients undergoing surgical treatment of anterior shoulder instability are unable to RTS.^1^^,^^35^^,^^55^^,^^80^

A recent study by Rossi et al showed that 74% of these patients failed to RTS because of reasons independent of the shoulder, such as fear for reinjury, kinesiophobia, and concerns about a new rehabilitation process.^72^ In addition, another study shows shoulder independent reasons such as self-motivation, social support, and shift in priorities as consideration not to RTS.^81^ Moreover, patients’ ability to RTS following surgical treatment of anterior shoulder instability is a multifactorial process. Recent insights show a relation between fear for shoulder movements (kinesiophobia) and return to preinjury level of sport after shoulder stabilizing surgery, suggesting a prominent reason not to RTS could be psychologically motivated and not solely shoulder function dependant.^85^

Numerous studies have evaluated and summarized quantitative variables regarding this subject.^1^^,^^35^^,^^55^ However, there is a lack of literature summarizing reasons for athletes who do not RTS following shoulder stabilizing surgery. Knowledge of these reasons may be helpful for both physicians and patients in the shared decision-making process and can possibly increase RTS rates following surgery. Therefore, the purpose of this study is to critically review patients’ reasons not to RTS after surgical treatment of anterior shoulder instability, comparing capsulolabral repair and bony reconstruction procedures. The hypothesis is that the mostly named reason for patients unable to RTS is not due to physical inability of the shoulder.

## Materials and methods

A review protocol was developed based on the Preferred Reporting Items for Systematic Reviews and Meta-Analysis (PRISMA) statement (www.prisma-statement.org) and was submitted to PROSPERO under review number CRD42022301102.

### Literature search

Relevant studies were identified by searching PubMed, Embase/Ovid, Cochrane Database of Systematic Reviews/Wiley, Cochrane Central Register of Controlled Trials/Wiley, SPORTDiscus/Ebsco, and Web of Science/Clarivate Analytics from inception up to October 19, 2021, and updated on August 11, 2022 (by T.P.I. and S.P.V., information specialist). The following terms, including synonyms and closely related words, were used as index terms or free-text words: ‘shoulder,’ ‘instability,’ ‘surgery,’ and ‘return to sports.’ Full search strategies for all databases are available as Supplementary Information (Supplementary Appendix S1). A language filter for English, German, and Dutch language was applied, when available in the database. Duplicate articles were excluded by the information specialist (S.P.V.) using EndNote X8 (2018; Analytics Clarivate, Philadelphia, PA, USA).

First, one reviewer (T.P.I.) extracted the references of the database search into the research software program (Rayyan QRCI, Cambridge, MA, USA).^61^ Then, two experienced reviewers (T.P.I. and S.H.S.) screened the studies based on abstract and title. Third, studies were screened based on full-text and included if they met the inclusion criteria. Any disagreement was resolved by discussion and consensus. If the authors were unable to reach a consensus, a final judgment was given by a third author L.P.E.V.

### Inclusion and exclusion criteria

Observational and interventional studies reporting reasons for patients not returning to sport after surgical treatment of shoulder instability were included. Articles were excluded 1) if they only stated reasons for return to a different level of sports, 2) if no reason for no RTS was mentioned, or 3) if the reasons were not assigned to a type of surgical treatment in case the article contained several types of surgery. Moreover, studies including patients with other types of ipsilateral shoulder surgery, animal studies, cadaveric studies, and abstract-only publications were excluded. When studies used the same cohort of patients, the study with the largest sample size was included.

### Data extraction

After quality appraisal, study data were extracted using a predetermined format. Baseline characteristics included study design, sample size, gender, data regarding surgical procedure(s), mean age at surgery, and mean age at follow-up. The primary outcome was reasons for patients not to RTS after surgical treatment of shoulder instability. No RTS was defined as failure to return to preinjury type of sport after surgery. Data were divided in either capsulolabral repair or bony reconstruction procedures. Capsulolabral repair was defined as any repair of the capsulolabral complex, both open and arthroscopic, which could be combined with a remplissage. Bony reconstruction procedures were defined as any reconstruction procedure using bony tissue (eg, Latarjet or allograft) to restore the stability of the shoulder. Besides these reasons, data were extracted regarding RTS, type of sports, and sports level. Data were extracted to Excel (Microsoft Excel 2016; Microsoft Corporation, Redmond, WA, USA).

### Quality appraisal and proportions

The quality of non-randomized studies was assessed using the Methodological Index for Non-Randomized Studies (MINORS) criteria.^77^ Assessment was performed by two authors T.P.I. and S.H.S. Any disagreement was resolved by discussion and consensus. If the authors were unable to reach a consensus, a final judgment was given by a third author (L.P.E.V). Quality assessment for randomized studies was assessed using the revised tool to assess risk of bias in randomized trials (RoB 2) developed by Cochrane.^79^ Proportions and 95% confidence intervals were calculated for each study and forest plots were generated using Excel to visualize the data.

## Results

### Screening and study characteristics

**Figure 1:**
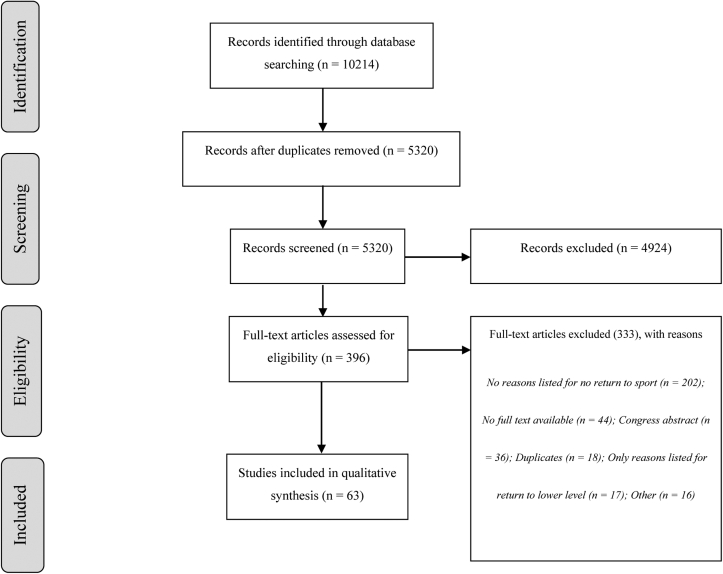
Flow diagram in/exclusion.

After removal of duplicates, 5320 studies were screened based on abstract and title (Fig. 1). Three-hundred-eighty-one studies underwent full-text screening, given that the abstract and title of 4939 studies did not match the inclusion criteria of this study. Of these 381 studies, 333 did not meet the inclusion criteria in the end, leaving 63 studies for inclusion, comprising 3545 patients.^3^, ^4^, ^5^^,^^8^^,^^10^, ^11^, ^12^^,^^14^, ^15^, ^16^, ^17^, ^18^, ^19^, ^20^^,^^22^^,^^24^, ^25^, ^26^^,^^28^, ^29^, ^30^, ^31^, ^32^, ^33^, ^34^^,^^36^, ^37^, ^38^, ^39^, ^40^^,^^42^^,^^45^, ^46^, ^47^, ^48^^,^^50^, ^51^, ^52^, ^53^, ^54^^,^^56^, ^57^, ^58^, ^59^, ^60^^,^^62^, ^63^, ^64^, ^65^^,^^67^, ^68^, ^69^, ^70^, ^71^, ^72^, ^73^, ^74^, ^75^^,^^81^^,^^84^^,^^86^^,^^88^, ^89^, ^90^ A flow chart diagram listing the reasons for exclusion is displayed in Figure 1. Forty-nine retrospective studies and ten prospective studies, of which one randomized controlled trial, were included.

Sixty-three studies listed reasons for no RTS after surgical treatment of shoulder instability, including a total of 3545 athletes.^3^, ^4^, ^5^^,^^8^^,^^10^, ^11^, ^12^^,^^14^, ^15^, ^16^, ^17^, ^18^, ^19^, ^20^^,^^22^^,^^24^, ^25^, ^26^^,^^28^, ^29^, ^30^, ^31^, ^32^, ^33^, ^34^^,^^36^, ^37^, ^38^, ^39^, ^40^^,^^42^^,^^45^, ^46^, ^47^, ^48^^,^^50^, ^51^, ^52^, ^53^, ^54^^,^^56^, ^57^, ^58^, ^59^, ^60^^,^^62^, ^63^, ^64^, ^65^^,^^67^, ^68^, ^69^, ^70^, ^71^, ^72^, ^73^, ^74^, ^75^^,^^81^^,^^84^^,^^86^^,^^88^, ^89^, ^90^ Study characteristics are displayed in Supplementary Appendix S2. Articles were published between 1991 and 2022, with sample sizes ranging from 16 to 208. The mean age for patients at the time of surgery ranged from 18 to 41.2 years and the mean follow-up ranged from 17 to 88.8 months.

### Quality assessment

The MINORS score ranged from 6 to 21 in studies listing reasons for patients not returning to sport after surgical treatment of shoulder instability (Supplementary Appendix S2). The RoB 2 score of the randomized study by Belangero, listing reasons for patients not returning to sport after surgical treatment of shoulder instability revealed a low risk of bias (Supplementary Appendix S2).^8^

### Reasons for patients not to return to sport following capsulolabral repair

**Figure 2:**
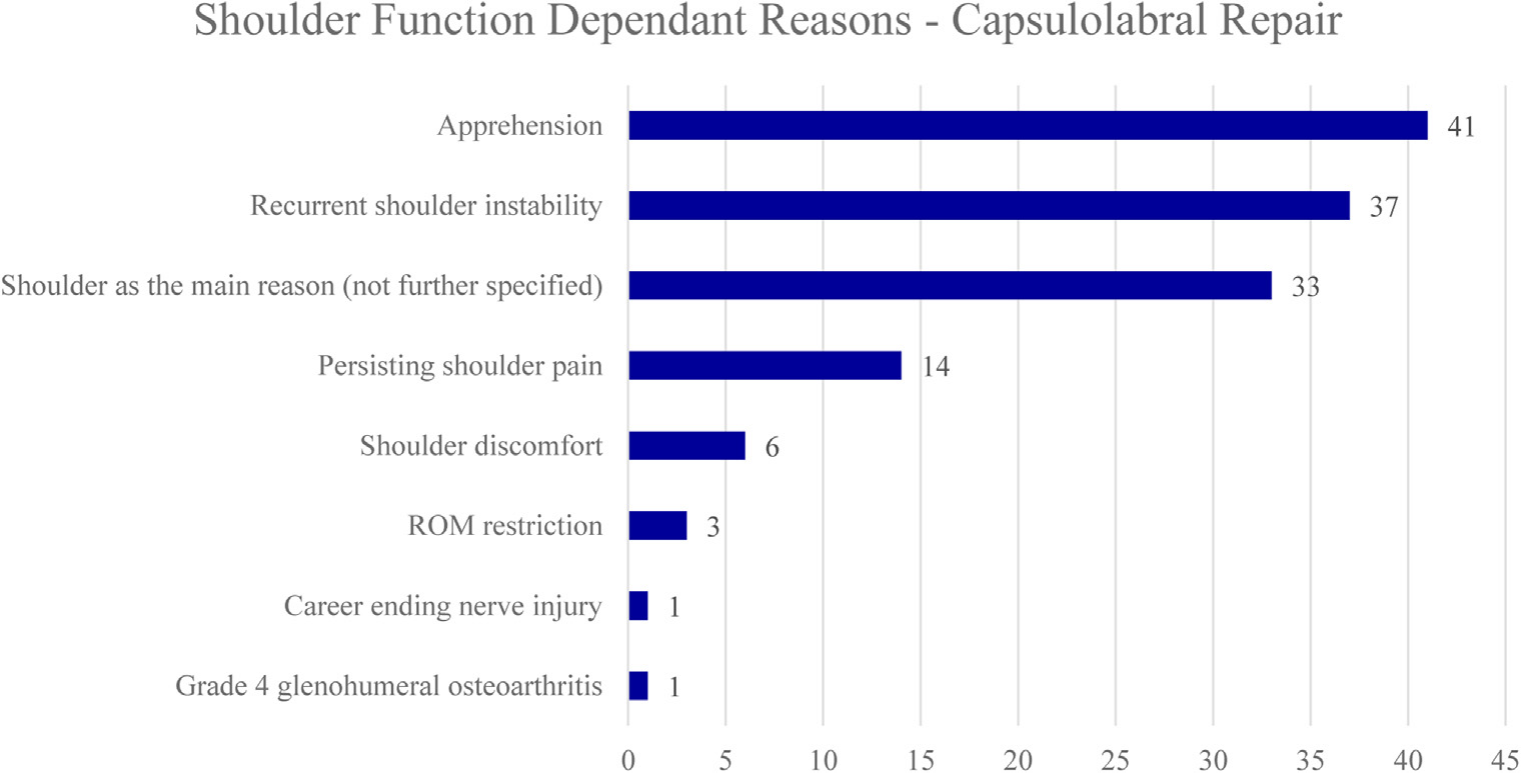
Shoulder function dependent reasons for no return to sports after capsulolabral repair. *ROM*, range of motion.

**Figure 3:**
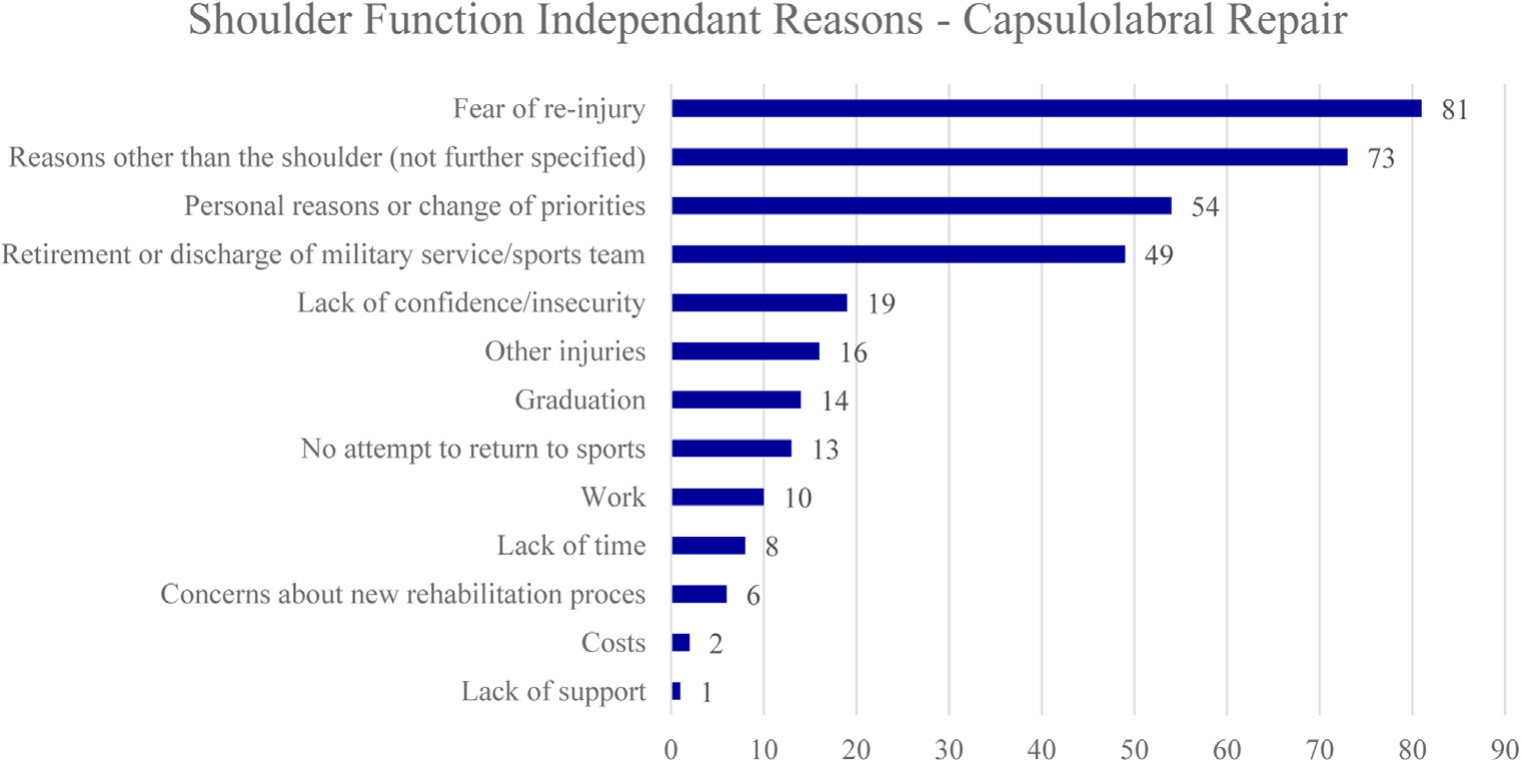
Shoulder function independent reasons for no return to sports after capsulolabral repair.

**Figure 4:**
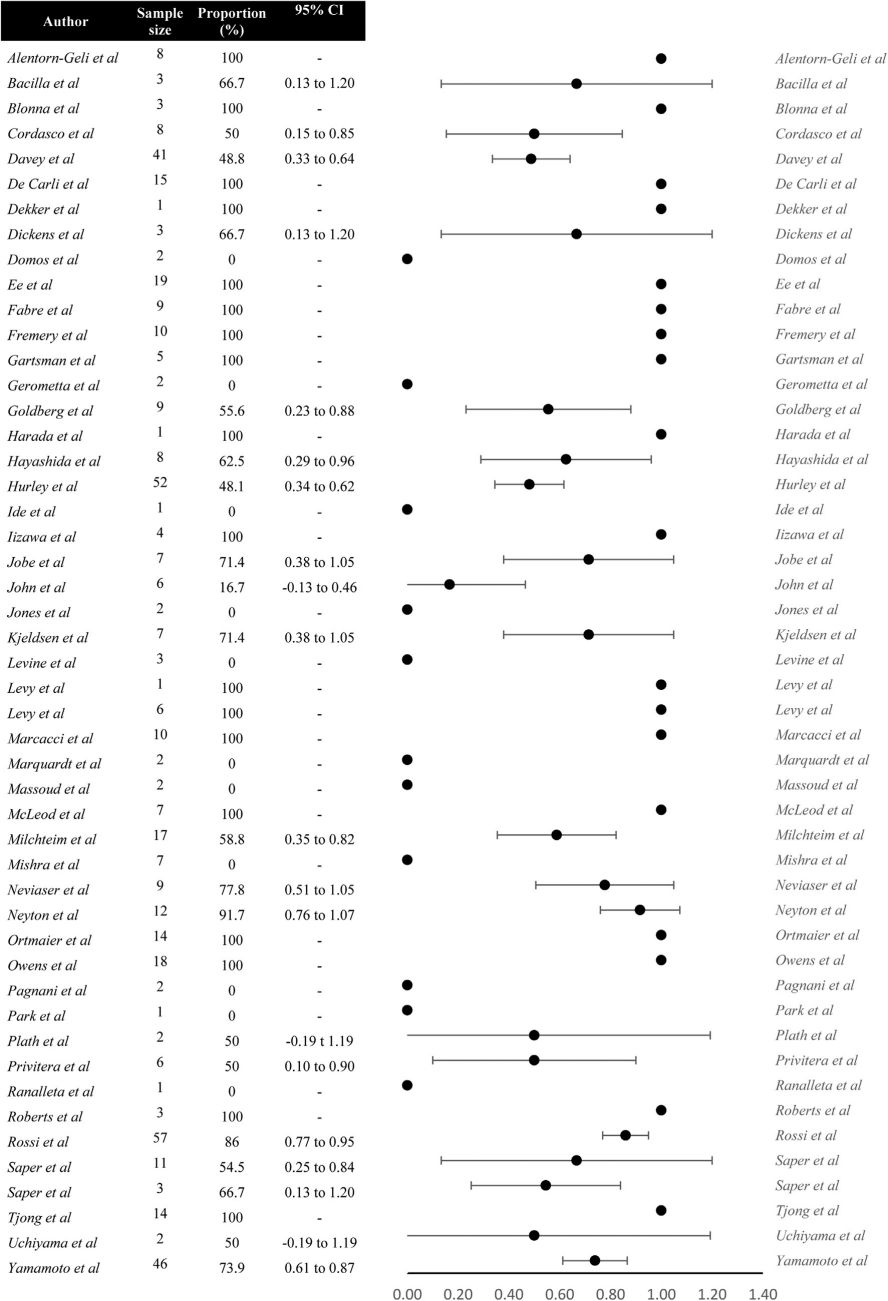
Forest plot demonstrating studies’ proportions of shoulder function independent reasons for no return to sport following capsulolabral repair. *CI*, confidence interval.

Of the 3545 athletes who were involved in sport prior to surgical treatment, 2588 (73%) athletes underwent capsulolabral repair, of which 482 (18%) did not RTS. When comparing different reasons for no RTS following capsulolabral repair, athletes cited shoulder function independent reasons more often than shoulder function dependent reasons (72% vs 28%). Mostly cited shoulder function dependent reasons for no RTS were apprehension (9%), recurrent shoulder instability (8%), and persisting shoulder pain (3%) (Fig. 2). Thirty-three athletes (7%) cited shoulder-related reasons which were not further specified. Mostly cited shoulder function independent reasons were fear of reinjury (17%), personal reasons or change of priorities (12%), and retirement/discharge from military service or sports team (10%) (Fig. 3). Seventy-three athletes (15%) cited shoulder unrelated reasons which were not further specified. Reasons for no RTS following capsulolabral repair are listed in Figures 2 and 3. The forest plot demonstrating shoulder function independent reasons not to RTS following capsulolabral repair showed proportions ranging from 0% to 100% (Fig. 4).

### Reasons for patients not to return to sport following bony reconstruction procedures

**Figure 5:**
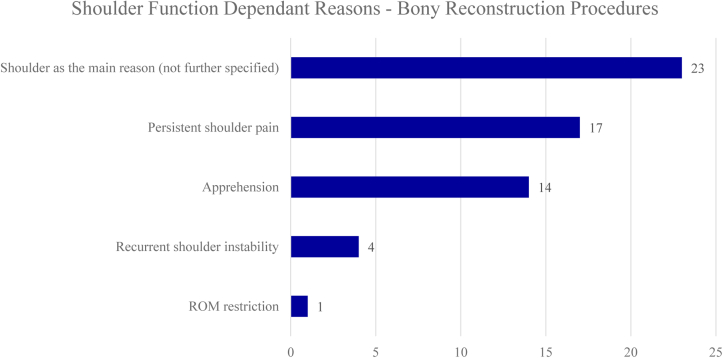
Shoulder function dependent reasons for no return to sports following bony reconstruction procedures. *ROM*, range of motion.

**Figure 6:**
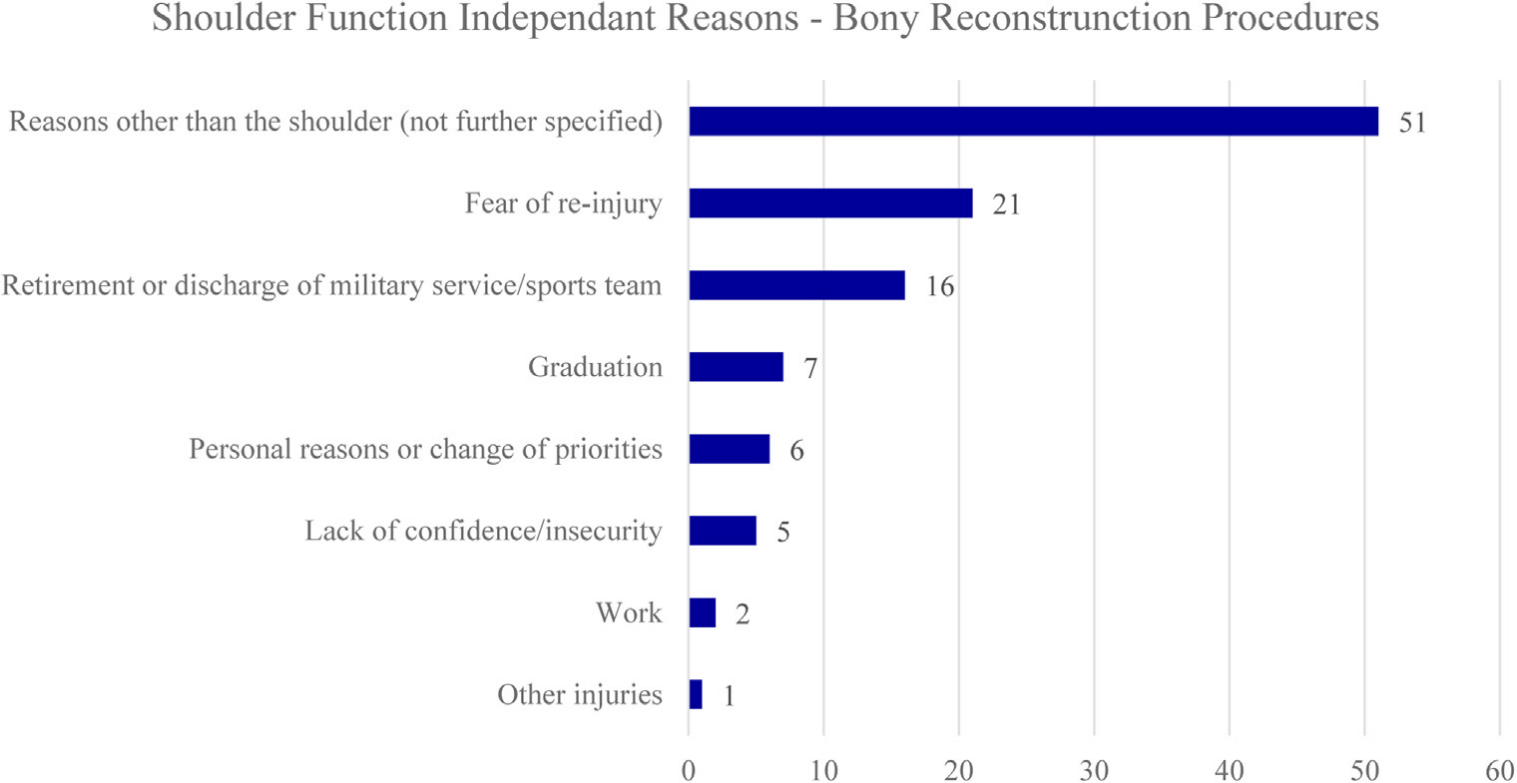
Shoulder function independent reasons for no return to sports following bony reconstruction procedures.

**Figure 7:**
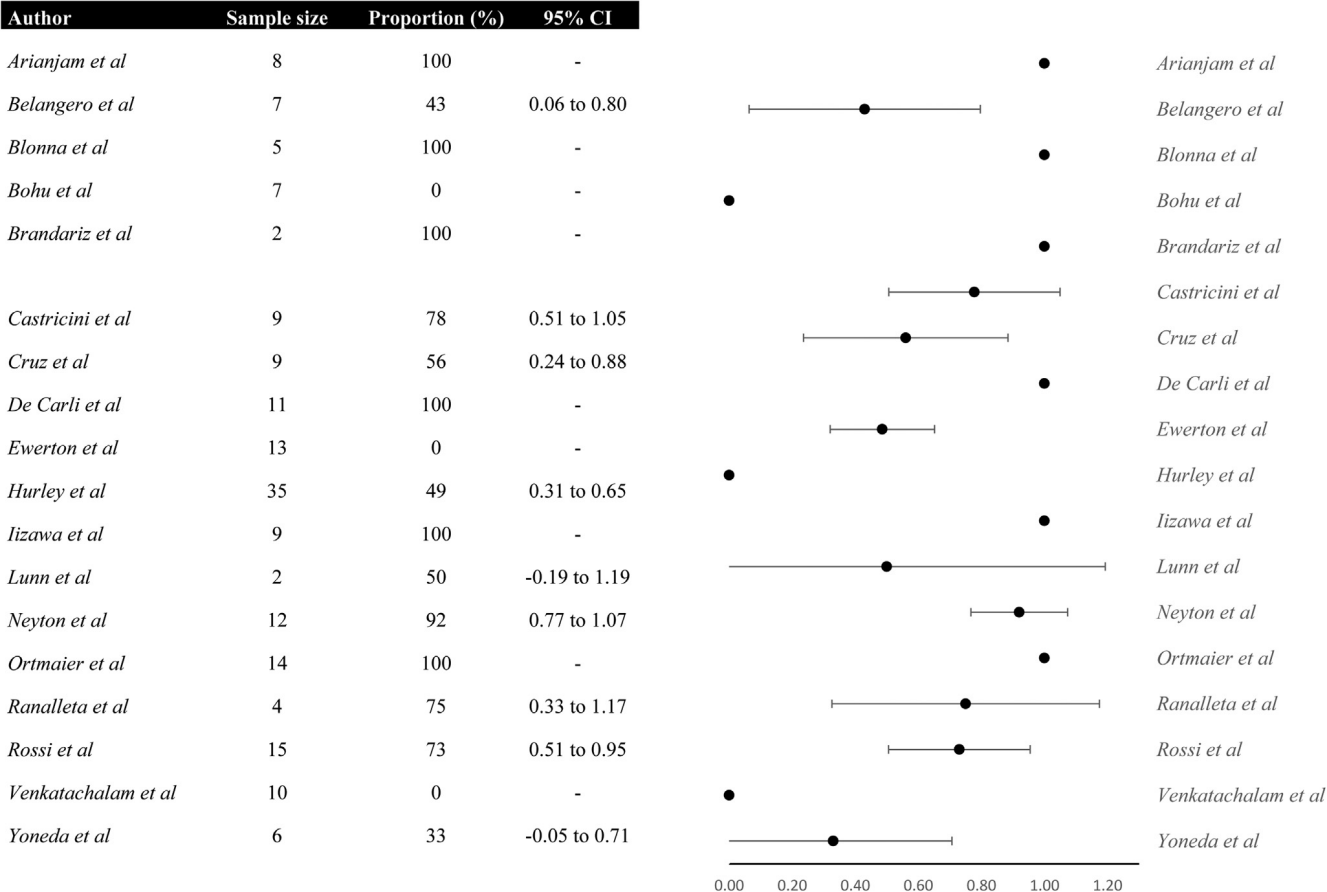
Forest plot demonstrating studies’ proportions of shoulder function independent reasons for no return to sport following bony reconstruction procedures. *CI*, confidence interval.

A total of 957 (27%) out of 3545 included athletes underwent surgical treatment with bony reconstruction procedures, of which 168 did not RTS (18%). Similar to capsulolabral repair, shoulder function independent reasons were cited more often compared to shoulder function dependent reasons following bony reconstruction procedures (65% vs 35%). Mostly cited shoulder function dependent reasons for not returning to sport were shoulder pain (10%), apprehension (8%), and recurrent shoulder instability (2%). Twenty-three athletes (13%) cited shoulder-related reasons which were not further specified. Mostly cited shoulder function independent reasons were fear of reinjury (13%), retirement/discharge from military service or sports team (10%) and graduation (4%). Fifty-one patients cited shoulder unrelated reasons which were not further specified (30%). Reasons for no RTS following bony reconstruction procedures are listed in Figures 5 and 6. The forest plot demonstrating shoulder function independent reasons not to RTS following bony reconstruction procedures showed proportions ranging from 0% to 100% (Fig. 7).

## Discussion

The purpose of this study was to critically and systematically review athletes’ reasons not to RTS after surgical treatment of anterior shoulder instability, in order to improve preoperative and postoperative patient counseling. The most important finding of this study is that majority of athletes who did not RTS following surgical treatment of shoulder instability did so due to shoulder function independent reasons, like fear of reinjury. The cause of this remains unclear. It might be associated with pathophysiological alterations such as functional cerebral changes fear or the unpredictability of an unstable shoulder.^49^^,^^76^ To elucidate this relationship, further (prospective) research is needed. Second, this study compared the reasons for patients who did not RTS following capsulolabral repair and bony reconstruction procedures. This study found a high failure rate to RTS for both treatments, which can be explained by the exclusion of studies with a 100% RTS rate. In addition, there were slight differences in the distribution of reasons not to RTS between the two types of surgical treatment, with shoulder function independent reasons seemingly being listed more often in patients undergoing capsulolabral repair.

### Return to sport

This systematic review showed that there are multiple reasons for athletes not to return to (their preinjury type of) sport, ranging from shoulder-related reasons (eg, recurrent shoulder instability and fear of reinjury) to shoulder unrelated reasons (eg, knee injury, motivation, and personal reasons). The maximal medical improvement following shoulder stabilization surgery may take up to 12 months, which could be explained by the underexposed psychological components of the issue.^66^ This can differ depending on the athletes’ motivational and type of medical and emotional support during the process. Despite the fact that the reported RTS rates after surgery seem high, returning to preinjury level of sports may be challenging. Two studies show that, respectively, 50% and 68% of the high-level athletes were able to RTS after surgical stabilization of the glenohumeral joint.^9^^,^^44^ Additionally, the study of Bak et al shows that after 4.5 years of follow-up, only 48% of their population was still participating in sports following surgical stabilization of the glenohumeral joint.^7^

### Psychological impact of physical trauma

In this study, 20% of the athletes who did not RTS after surgical treatment of shoulder instability cited fear of reinjury, lack of confidence, or insecurity as reason to cease their sport activity. Psychological components have been reported to play a key role in considering the RTS of athletes.^43^^,^^72^^,^^81^ Kordasiewicz et al found that 46% of the patients were anxious to RTS after undergoing the Latarjet procedure, which is regarded as the type of shoulder stabilizing procedure yielding the lowest prevalence of recurrent instability.^1^ In addition, a qualitative study containing 25 semi-structured interviews with shoulder instability patients also revealed that reasons which were not a physical shoulder problem (eg, fear of reinjury, social support, and self-motivation) greatly influenced the patients’ decision to RTS.^81^ Although recent studies already showed that kinesiophobia is correlated to the RTS after shoulder stabilization surgery, there is no (p)rehabilitation consensus protocol currently available which includes this psychosocial component.^85^ Future studies are needed to reach consensus about the content of such a (p)rehabilitation protocol, explicitly including the psychological component of the physical problem of shoulder instability.

### Return to sport as outcome measure

Patients often have high expectations of surgical treatment of shoulder instability.^83^ One of those key expectations is RTS. However, to date, there is no consensus on the definition of RTS in current literature, which may result in heterogeneity, potentially causing bias among studies investigating RTS.^21^^,^^92^ Most studies define RTS as the return to any level of sport postoperatively. However, others strictly define it as the return to preinjury level of sport, which was defined as successful RTS in this study. Moreover, there is also no consensus on what the RTS includes. Some studies refer to return to competitive play or competing a game, while other studies also include return to practice. Furthermore, it is not well-defined for what period of time an athlete should return for it to be defined as RTS and whether or not physical complaints are allowed to be present during this return. Broad consensus is needed to clearly define these dimensions to reduce potential bias.

Moreover, this study showed that the majority of athletes who did not RTS following surgical treatment of shoulder instability did so due to shoulder function independent reasons, including reasons like fear of reinjury and apprehension. Almost all included studies did not report on readiness to RTS, which could explain the high amount of shoulder function independent reasons. If an athlete is not ready to RTS, it might not be fair to assess the surgical treatment with RTS as an outcome.

### Limitations

This systematic review has several limitations. First, most (53/63) included studies had a retrospective design, which may introduce bias into the design of a study, such as recall bias.^3^^,^^4^^,^^10^^,^^12^^,^^14^^,^^16^, ^17^, ^18^, ^19^^,^^22^^,^^24^^,^^25^^,^^29^, ^30^, ^31^, ^32^, ^33^, ^34^^,^^37^, ^38^, ^39^, ^40^^,^^45^, ^46^, ^47^, ^48^^,^^50^, ^51^, ^52^, ^53^, ^54^^,^^56^, ^57^, ^58^, ^59^, ^60^^,^^62^, ^63^, ^64^, ^65^^,^^67^, ^68^, ^69^, ^70^, ^71^, ^72^, ^73^, ^74^, ^75^^,^^81^^,^^84^^,^^86^^,^^88^, ^89^, ^90^ Moreover, retrospective studies are susceptible to methodological flaws such as selection bias and possible loss of data.^82^ Second, many studies reported whether reasons for not returning to sports after surgery were shoulder related or not shoulder related, but reasons were not always specified. This precluded a detailed statement of the nature of the reasons and if they were largely physically or psychologically motivated, potentially explaining the wide spectrum of proportions in the forest plots. In addition, RTS rate in general is a relevant and important patient-centered outcome measure to assess the treatment chain. However, it may not be an optimal outcome to evaluate the effect of surgery itself. Both physical and psychological rehabilitation remain of vital importance. Third, this study included studies ranging from 1991 to 2022, with nine out of 63 studies being published before 2000. The main focus was to include as many reasons of patients not returning to sport, not specifically focusing on one group, for instance professional athletes. This could potentially lead to high heterogeneity and could create bias. However, subgroup analysis was performed comparing studies only including competitive and professional athletes, and results were comparable to those of the entire included population in this systematic review.

Further research should focus on the integration of these two aspects within the treatment protocol. Recently Kim et al published a comparable study investigating reasons for failure to sports after arthroscopic Bankart repair in 17 studies containing 813 athletes.^41^ In contrast to that study, this study managed to summarize data of 63 studies containing reasons of 3545 athletes ceasing sports activity following both capsulolabral repair and bony reconstruction procedures, making it the largest sample size currently available regarding this subject.

## Conclusion

The majority of athletes who did not RTS following surgical treatment for anterior shoulder instability did so due to shoulder function independent reasons, such as fear of reinjury. However, there was a high variety between studies and many reasons were unspecified, warranting unified definitions for reasons of patients that do not RTS.

## Disclaimers

Funding: No funding was disclosed by the authors.

Conflicts of interest: The authors, their immediate families, and any research foundation with which they are affiliated have not received any financial payments or other benefits from any commercial entity related to the subject of this article.

## References

[1] Abdul-Rassoul H., Galvin J.W., Curry E.J., Simon J., Li X. (2019). Return to sport after surgical treatment for anterior shoulder instability: a systematic review. Am J Sports Med.

[2] Adam M., Attia A.K., Alhammoud A., Aldahamsheh O., Al Ateeq Al Dosari M., Ahmed G. (2018). Arthroscopic Bankart repair for the acute anterior shoulder dislocation: systematic review and meta-analysis. Int Orthop.

[3] Alentorn-Geli E., Álvarez-Díaz P., Doblas J., Steinbacher G., Seijas R., Ares O. (2016). Return to sports after arthroscopic capsulolabral repair using knotless suture anchors for anterior shoulder instability in soccer players: minimum 5-year follow-up study. Knee Surg Sports Traumatol Arthrosc.

[4] Arianjam A., Bell S.N., Coghlan J., Old J., Sloan R. (2015). Outcomes for intra-substance free coracoid graft in patients with antero-inferior instability and glenoid bone loss in a population of high-risk athletes at a minimum follow-up of 2 years. Shoulder Elbow.

[5] Bacilla P., Field L.D., Savoie F.H. (1997). Arthroscopic Bankart repair in a high demand patient population. Arthroscopy.

[6] Bah A., Lateur G.M., Kouevidjin B.T., Bassinga J.Y.S., Issa M., Jaafar A. (2018). Chronic anterior shoulder instability with significant Hill-Sachs lesion: arthroscopic Bankart with remplissage versus open Latarjet procedure. Orthop Traumatol Surg Res.

[7] Bak K., Spring B.J., Henderson J.P. (2000). Inferior capsular shift procedure in athletes with multidirectional instability based on isolated capsular and ligamentous redundancy. Am J Sports Med.

[8] Belangero P.S., Lara P.H.S., Figueiredo E.A., Andreoli C.V., de Castro Pochini A., Ejnisman B. (2021). Bristow versus Latarjet in high-demand athletes with anterior shoulder instability: a prospective randomized comparison. JSES Int.

[9] Bigliani L.U., Kurzweil P.R., Schwartzbach C.C., Wolfe I.N., Flatow E.L. (1994). Inferior capsular shift procedure for anterior-inferior shoulder instability in athletes. Am J Sports Med.

[10] Blonna D., Bellato E., Caranzano F., Assom M., Rossi R., Castoldi F. (2016). Arthroscopic Bankart repair versus open Bristow-Latarjet for shoulder instability: a matched-pair multicenter study focused on return to sport. Am J Sports Med.

[11] Bohu Y., Klouche S., Gerometta A., Herman S., Lefevre N. (2016). Outpatient Latarjet surgery for gleno-humeral instability: prospective comparative assessment of feasibility and safety. Orthop Traumatol Surg Res.

[12] Brandariz R.N., Gorodischer T.D., Pasqualini I., Rossi L.A., Tanoira I., Ranalletta M. (2021). The Latarjet procedure without remplissage is effective to restore stability in athletes with glenoid bone defects greater than 25% and off-track Hill-Sachs lesions. Arthroscopy.

[13] Cameron K.L., Mauntel T.C., Owens B.D. (2017). The epidemiology of glenohumeral joint instability: incidence, burden, and long-term consequences. Sports Med Arthrosc Rev.

[14] Castricini R., Castioni D., De Benedetto M., Cimino M., Massarini A., Galasso O. (2022). Arthroscopic Latarjet for primary shoulder instability with off-track lesions or revision surgery yields satisfactory clinical results and reliable return to sport and work at minimum 3-year follow-up: a single surgeon's experience on 95 cases. Arthroscopy.

[15] Cordasco F.A., Lin B., Heller M., Asaro L.A., Ling D., Calcei J.G. (2020). Arthroscopic shoulder stabilization in the young athlete: return to sport and revision stabilization rates. J Shoulder Elbow Surg.

[16] Cruz C.A., Sy J., Miles R., Bottoni C.R., Min K.S. (2021). Surgical treatment of anterior shoulder instability with glenoid bone loss with the Latarjet procedure in active-duty military service members. J Shoulder Elbow Surg.

[17] Davey M.S., Hurley E.T., Gaafar M., Mullett H., Pauzenberger L. (2021). Arthroscopic Bankart repair for primary versus recurrent anterior instability in athletes results in excellent clinical outcomes, high rates of return to play, and low recurrence rates. Arthrosc Sports Med Rehabil.

[18] De Carli A., Vadalà A., Proietti L., Ponzo A., Desideri D., Ferretti A. (2019). Latarjet procedure versus open capsuloplasty in traumatic anterior shoulder dislocation: long-term clinical and functional results. Int Orthop.

[19] Dekker T.J., Goldenberg B., Lacheta L., M P.H., Millett P.J. (2020). Anterior shoulder instability in the professional athlete: return to competition, time to return, and career length. Orthop J Sports Med.

[20] Dickens J.F., Rue J.P., Cameron K.L., Tokish J.M., Peck K.Y., Allred C.D. (2017). Successful return to sport after arthroscopic shoulder stabilization versus nonoperative management in contact athletes with anterior shoulder instability: a prospective multicenter study. Am J Sports Med.

[21] Doege J., Ayres J.M., Mackay M.J., Tarakemeh A., Brown S.M., Vopat B.G. (2021). Defining return to sport: a systematic review. Orthopaedic J Sports Med.

[22] Domos P., Ascione F., Wallace A.L. (2019). Arthroscopic Bankart repair with remplissage for non-engaging Hill-Sachs lesion in professional collision athletes. Shoulder Elbow.

[23] Dutton M., Tam N., Gray J. (2019). Incidence and impact of time loss and non-time-loss shoulder injury in elite South African cricketers: a one-season, prospective cohort study. J Sci Med Sport.

[24] Ee G.W., Mohamed S., Tan A.H. (2011). Long term results of arthroscopic Bankart repair for traumatic anterior shoulder instability. J Orthop Surg Res.

[25] Fabre T., Abi-Chahla M.L., Billaud A., Geneste M., Durandeau A. (2010). Long-term results with Bankart procedure: a 26-year follow-up study of 50 cases. J Shoulder Elbow Surg.

[26] Fremerey R., Bosch U., Lobenhoffer P., Wippermann B. (2006). Joint position awareness and sports activity after capsulolabral reconstruction in the overhead athlete. Int J Sports Med.

[27] Galvin J.W., Ernat J.J., Waterman B.R., Stadecker M.J., Parada S.A. (2017). The epidemiology and natural history of anterior shoulder instability. Curr Rev Musculoskelet Med.

[28] Gartsman G.M., Roddey T.S., Hammerman S.M. (2000). Arthroscopic treatment of anterior-inferior glenohumeral instability. Two to five-year follow-up. J Bone Joint Surg Am.

[29] Gerometta A., Rosso C., Klouche S., Hardy P. (2016). Arthroscopic Bankart shoulder stabilization in athletes: return to sports and functional outcomes. Knee Surg Sports Traumatol Arthrosc.

[30] Goldberg B.J., Nirschl R.P., McConnell J.P., Pettrone F.A. (1993). Arthroscopic transglenoid suture capsulolabral repairs: preliminary results. Am J Sports Med.

[31] Harada Y., Iwahori Y., Kajita Y., Takahashi R., Yokoya S., Sumimoto Y. (2021). Return to sports after arthroscopic Bankart repair on the dominant shoulder in overhead athletes. J Orthop Sci.

[32] Hayashida K., Yoneda M., Mizuno N., Fukushima S., Nakagawa S. (2006). Arthroscopic Bankart repair with knotless suture anchor for traumatic anterior shoulder instability: results of short-term follow-up. Arthroscopy.

[33] Hurley E.T., Davey M.S., Mojica E.S., Montgomery C., Gaafar M., Jazrawi L.M. (2021). Analysis of patients unable to return to play following arthroscopic Bankart repair. Surgeon.

[34] Hurley E.T., Davey M.S., Montgomery C., Moore D.M., Mojica E.S., Gaafar M. (2022). Analysis of athletes who did not return to play after open Latarjet. Orthop J Sports Med.

[35] Hurley E.T., Montgomery C., Jamal M.S., Shimozono Y., Ali Z., Pauzenberger L. (2019). Return to play after the Latarjet procedure for anterior shoulder instability: a systematic review. Am J Sports Med.

[36] Ide J., Maeda S., Takagi K. (2004). Arthroscopic Bankart repair using suture anchors in athletes: patient selection and postoperative sports activity. Am J Sports Med.

[37] Iizawa N., Yoneda M., Yamada S., Mizuno N., Goto K., Iwashita S. (2020). Benefits of bone graft augmentation to arthroscopic Bankart repair for recurrent anterior shoulder instability with glenoid bone loss. Knee Surg Sports Traumatol Arthrosc.

[38] Jobe F.W., Giangarra C.E., Kvitne R.S., Glousman R.E. (1991). Anterior capsulolabral reconstruction of the shoulder in athletes in overhand sports. Am J Sports Med.

[39] John M., Nebelung W., Röpke M., Ender S.A., Urbach D. (2007). Arthroscopic labrum reconstruction with capsular shift in anterior shoulder instability: improved midterm results by using a standardized suprabicipital camera position. Arthroscopy.

[40] Jones K.J., Kahlenberg C.A., Dodson C.C., Nam D., Williams R.J., Altchek D.W. (2012). Arthroscopic capsular plication for microtraumatic anterior shoulder instability in overhead athletes. Am J Sports Med.

[41] Kim M., Haratian A., Fathi A., Kim D.R., Patel N., Bolia I.K. (2022). Can we identify why athletes fail to return to sports after arthroscopic Bankart repair: a systematic review and meta-analysis. Am J Sports Med.

[42] Kjeldsen S.R., Tordrup P.J., Hvidt E.P. (1996). Return to sport after a Bankart operation of the shoulder using the Mitek anchor system. Scand J Med Sci Sports.

[43] Kordasiewicz B., Małachowski K., Kicinski M., Chaberek S., Boszczyk A., Marczak D. (2019). Intraoperative graft-related complications are a risk factor for recurrence in arthroscopic Latarjet stabilisation. Knee Surg Sports Traumatol Arthrosc.

[44] Kvitne R.S., Jobe F.W., Jobe C.M. (1995). Shoulder instability in the overhand or throwing athlete. Clin Sports Med.

[45] Levine W.N., Arroyo J.S., Pollock R.G., Flatow E.L., Bigliani L.U. (2000). Open revision stabilization surgery for recurrent anterior glenohumeral instability. Am J Sports Med.

[46] Levy D.M., Gvozdyev B.V., Schulz B.M., Boselli K.J., Ahmad C.S. (2014). Arthroscopic anterior shoulder stabilization with percutaneous assistance and posteroinferior capsular plication. Am J Orthop (Belle Mead NJ).

[47] Levy O., Matthews T., Even T. (2007). The "purse-string" technique: an arthroscopic technique for stabilization of anteroinferior instability of the shoulder with early and medium-term results. Arthroscopy.

[48] Lima E.B.S., Osés G.L., de Godoy G.P., Lara P.H.S., Ribeiro L.M., de Figueiredo E.A. (2022). Evaluation of Latarjet procedure in female athletes: a 3-year follow-up prospective cohort study. JSES Int.

[49] Livett M.F., Williams D., Potter H., Cairns M. (2021). Functional cortical changes associated with shoulder instability – a systematic review. Shoulder & Elbow.

[50] Lunn J.V., Castellano-Rosa J., Walch G. (2008). Recurrent anterior dislocation after the Latarjet procedure: outcome after revision using a modified Eden-Hybinette operation. J Shoulder Elbow Surg.

[51] Marcacci M., Zaffagnini S., Petitto A., Neri M.P., Iacono F., Visani A. (1996). Arthroscopic management of recurrent anterior dislocation of the shoulder: analysis of technical modifications on the Caspari procedure. Arthroscopy.

[52] Marquardt B., Pötzl W., Witt K.A., Steinbeck J. (2005). A modified capsular shift for atraumatic anterior-inferior shoulder instability. Am J Sports Med.

[53] Massoud S.N., Levy O., Copeland S.A. (2002). The vertical-apical suture Bankart lesion repair for anteroinferior glenohumeral instability. J Shoulder Elbow Surg.

[54] McLeod A., Delaney R. (2022). Outcomes of the arthroscopic Bankart procedure in Irish collision sport athletes. Ir J Med Sci.

[55] Memon M., Kay J., Cadet E.R., Shahsavar S., Simunovic N., Ayeni O.R. (2018). Return to sport following arthroscopic Bankart repair: a systematic review. J Shoulder Elbow Surg.

[56] Milchteim C., Tucker S.A., Nye D.D., Lamour R.J., Liu W., Andrews J.R. (2016). Outcomes of Bankart repairs using modern arthroscopic technique in an athletic population. Arthroscopy.

[57] Mishra A., Sharma P., Chaudhary D. (2012). Analysis of the functional results of arthroscopic Bankart repair in posttraumatic recurrent anterior dislocations of shoulder. Indian J Orthop.

[58] Neviaser R.J., Benke M.T., Neviaser A.S. (2017). Mid-term to long-term outcome of the open Bankart repair for recurrent traumatic anterior dislocation of the shoulder. J Shoulder Elbow Surg.

[59] Neyton L., Young A., Dawidziak B., Visona E., Hager J.P., Fournier Y. (2012). Surgical treatment of anterior instability in rugby union players: clinical and radiographic results of the Latarjet-Patte procedure with minimum 5-year follow-up. J Shoulder Elbow Surg.

[60] Ortmaier R., Fink C., Schobersberger W., Kindermann H., Mattiassich G., Hochreiter J. (2019). Return to sports after glenoid reconstruction using an implant-free iliac crest bone graft. Orthop Traumatol Surg Res.

[61] Ouzzani M., Hammady H., Fedorowicz Z., Elmagarmid A. (2016). Rayyan—a web and mobile app for systematic reviews. Syst Rev.

[62] Owens B.D., DeBerardino T.M., Nelson B.J., Thurman J., Cameron K.L., Taylor D.C. (2009). Long-term follow-up of acute arthroscopic Bankart repair for initial anterior shoulder dislocations in young athletes. Am J Sports Med.

[63] Pagnani M.J., Dome D.C. (2002). Surgical treatment of traumatic anterior shoulder instability in American football players. J Bone Joint Surg Am.

[64] Pagnani M.J., Warren R.F., Altchek D.W., Wickiewicz T.L., Anderson A.F. (1996). Arthroscopic shoulder stabilization using transglenoid sutures. A four-year minimum followup. Am J Sports Med.

[65] Park I., Park C.J., Lee J.H., Hyun H.S., Park J.Y., Shin S.J. (2018). Clinical outcomes and recurrence rates after arthroscopic stabilization procedures in young patients with a glenoid bone Erosion: a comparative study between glenoid Erosion more and less than 20. Arthroscopy.

[66] Patel B.H., Lu Y., Agarwalla A., Puzzitiello R.N., Nwachukwu B.U., Cvetanovich G.L. (2020). Maximal medical improvement following shoulder stabilization surgery may require up to 1 year: a systematic review. HSS J.

[67] Plath J.E., Feucht M.J., Bangoj R., Martetschläger F., Wörtler K., Seppel G. (2015). Arthroscopic suture anchor fixation of bony Bankart lesions: clinical outcome, magnetic resonance imaging results, and return to sports. Arthroscopy.

[68] Privitera D.M., Bisson L.J., Marzo J.M. (2012). Minimum 10-year follow-up of arthroscopic intra-articular Bankart repair using bioabsorbable tacks. Am J Sports Med.

[69] Ranalletta M., Rossi L.A., Bertona A., Tanoira I., Hidalgo I.A., Maignon G.D. (2018). Modified Latarjet without capsulolabral repair in rugby players with recurrent anterior glenohumeral instability and significant glenoid bone loss. Am J Sports Med.

[70] Ranalletta M., Rossi L.A., Sirio A., Diaz Dilernia F., Bertona A., Maignon G.D. (2017). Return to sports and recurrences after arthroscopic anterior shoulder stabilization in martial arts athletes. Orthop J Sports Med.

[71] Roberts S.N., Taylor D.E., Brown J.N., Hayes M.G., Saies A. (1999). Open and arthroscopic techniques for the treatment of traumatic anterior shoulder instability in Australian rules football players. J Shoulder Elbow Surg.

[72] Rossi L.A., Tanoira I., Brandariz R., Pasqualini I., Ranalletta M. (2021). Reasons why athletes do not return to sports after arthroscopic Bankart repair: a comparative study of 208 athletes with minimum 2-year follow-up. Orthop J Sports Med.

[73] Rossi L.A., Tanoira I., Gorodischer T., Pasqualini I., Muscolo D.L., Ranalletta M. (2020). Are the classic and the congruent arc Latarjet procedures equally effective for the treatment of recurrent shoulder instability in athletes?. Am J Sports Med.

[74] Saper M.G., Courson J., Milchteim C., Plummer H., Andrews J.R., Ostrander R.V. (2021). Successful outcomes and return to sport after arthroscopic Bankart repair in National Collegiate Athletic Association and National Football League football players. Clin J Sport Med.

[75] Saper M.G., Milchteim C., Zondervan R.L., Andrews J.R., Ostrander R.V. (2017). Outcomes after arthroscopic Bankart repair in adolescent athletes participating in collision and contact sports. Orthop J Sports Med.

[76] Shitara H., Shimoyama D., Sasaki T., Hamano N., Ichinose T., Yamamoto A. (2015). The neural correlates of shoulder apprehension: a functional MRI study. PLoS One.

[77] Slim K., Nini E., Forestier D., Kwiatkowski F., Panis Y., Chipponi J. (2003). Methodological index for non-randomized studies (minors): development and validation of a new instrument. ANZ J Surg.

[78] Steinmetz R.G., Guth J.J., Matava M.J., Brophy R.H., Smith M.V. (2022). Return to play following nonsurgical management of Superior labrum anterior-posterior tears: a systematic review. J Shoulder Elbow Surg.

[79] Sterne J.A.C., Savović J., Page M.J., Elbers R.G., Blencowe N.S., Boutron I. (2019). RoB 2: a revised tool for assessing risk of bias in randomised trials. BMJ.

[80] Thayaparan A., Yu J., Horner N.S., Leroux T., Alolabi B., Khan M. (2019). Return to sport after arthroscopic superior labral anterior-posterior repair: a systematic review. Sports Health.

[81] Tjong V.K., Devitt B.M., Murnaghan M.L., Ogilvie-Harris D.J., Theodoropoulos J.S. (2015). A qualitative investigation of return to sport after arthroscopic Bankart repair: beyond stability. Am J Sports Med.

[82] Tofthagen C. (2012). Threats to validity in retrospective studies. J Adv Pract Oncol.

[83] Trojan J.D., DeFroda S.F., Mulcahey M.K. (2019). Patient understanding, expectations, outcomes, and satisfaction regarding surgical management of shoulder instability. Phys Sportsmed.

[84] Uchiyama Y., Hamada K., Miyazaki S., Handa A., Fukuda H. (2009). Neer modified inferior capsular shift procedure for recurrent anterior instability of the shoulder in judokas. Am J Sports Med.

[85] Vascellari A., Ramponi C., Venturin D., Ben G., Coletti N. (2019). The relationship between kinesiophobia and return to sport after shoulder surgery for recurrent anterior instability. Joints.

[86] Venkatachalam S., Storey P., Macinnes S.J., Ali A., Potter D. (2016). The Sheffield bone block procedure: a new operation for the treatment of glenoid bone loss in patients with anterior traumatic shoulder instability. Shoulder Elbow.

[87] Virta L., Joranger P., Brox J.I., Eriksson R. (2012). Costs of shoulder pain and resource use in primary health care: a cost-of-illness study in Sweden. BMC Musculoskelet Disord.

[88] Yamamoto N., Kijima H., Nagamoto H., Kurokawa D., Takahashi H., Sano H. (2015). Outcome of Bankart repair in contact versus non-contact athletes. Orthop Traumatol Surg Res.

[89] Yee A.J., Devane P.A., Horne G. (1999). Surgical repair for recurrent anterior instability of the shoulder. Aust N Z J Surg.

[90] Yoneda M., Hayashida K., Wakitani S., Nakagawa S., Fukushima S. (1999). Bankart procedure augmented by coracoid transfer for contact athletes with traumatic anterior shoulder instability. Am J Sports Med.

[91] Zacchilli M.A., Owens B.D. (2010). Epidemiology of shoulder dislocations presenting to emergency departments in the United States. J Bone Joint Surg Am.

[92] Zaremski J.L., Galloza J., Sepulveda F., Vasilopoulos T., Micheo W., Herman D.C. (2017). Recurrence and return to play after shoulder instability events in young and adolescent athletes: a systematic review and meta-analysis. Br J Sports Med.

