# Supplementary material for: Why do patients with anterior shoulder instability not return to sport after surgery? A systematic review of 63 studies comprising 3545 patients
Source: JSES Int. 2023 Jan 20;7(3):376–84. doi: 10.1016/j.jseint.2023.01.001 (PMC10229421; doi:10.1016/j.jseint.2023.01.001)
Supplement: Supplementary Appendix S1 [file mmc1.docx]

**Supplement 1: Search Strategy**

*Searching and deduplication were performed on October 19th 2021* and rerun on August 11th 2022. The number of hits per line are based on the update in August*. No filters were applied.*

**PubMed**

| Search | Query | Results |
| --- | --- | --- |
| #4 | Search: #1 AND #2 AND #3 Sort by: Most Recent | 3,069 |
| #3 | Search: ("Return to Sport"[Mesh] OR ((resum*[tiab] OR return*[tiab] OR continu*[tiab] OR discontinu*[tiab]) AND (sport*[tiab] OR play*[tiab] OR activit*[tiab] OR competit*[tiab] OR function*[tiab] OR "Athletes"[Mesh] OR athlete*[tiab]))) Sort by: Most Recent | 460,090 |
| #2 | Search: ("surgery" [Subheading] OR "Surgical Procedures, Operative"[Mesh] OR "Surgeons"[Mesh] OR surger*[tiab] OR surgic*[tiab] OR surgeon*[tiab] OR operation*[tiab] OR operative*[tiab] OR invasive*[tiab] OR restorati*[tiab] OR reconstruct*[tiab] OR bony[tiab] OR Bankart[tiab] OR "Bankart Lesions/surgery"[Mesh] OR SLAP*[tiab] OR superior–labr*[tiab] OR (arthroscopic[tiab] AND stabili*[tiab]) OR latarjet[tiab]) Sort by: Most Recent | 5,761,368 |
| #1 | Search: ("Shoulder Dislocation"[Mesh] OR "Shoulder Injuries"[Mesh] OR (("Shoulder"[Mesh] OR "Shoulder Joint"[Mesh] OR shoulder*[tiab] OR glenohumeral[tiab] OR labr*[tiab]) AND ("Joint Dislocations"[Mesh] OR "Joint Instability"[Mesh] OR dislocat*[tiab] OR luxat*[tiab] OR subluxat*[tiab] OR instabilit*[tiab] OR unstable[tiab] OR instable[tiab] OR injur*[tiab] OR "Pain"[Mesh] OR pain[tiab]))) Sort by: Most Recent | 55,732 |

**Embase/Ovid**

| 1 | exp shoulder/ or (shoulder* or glenohumeral or labr*).ti,ab,kw. | 164205 |
| --- | --- | --- |
| 2 | exp joint dislocation/ or exp joint instability/ or exp pain/ or (dislocat* or luxat* or subluxat* or instabilit* or unstable or instable or injur* or pain).ti,ab,kw. | 3313884 |
| 3 | exp shoulder dislocation/ or exp recurrent shoulder dislocation/ or exp shoulder injury/ or (1 and 2) | 84213 |
| 4 | su.fs. or exp surgical technique/ or exp shoulder surgery/ or exp surgeon/ or exp Bankart lesion/su or (surger* or surgic* or surgeon* or operation* or operative* or invasive* or restorati* or reconstruct* or bony or Bankart or SLAP* or superior-labr* or (arthroscopic and stabili*) or Latarjet).ti,ab,kw. | 6427265 |
| 5 | exp return to sport/ or ((resum* or return* or continu* or discontinu*).ti,ab,kw. and (exp athlete/ or (sport* or play* or activit* or competit*or function* or athlete*).ti,ab,kw.)) | 385519 |
| 6 | 3 and 4 and 5 | 3143 |

**Cochrane Database of Systematic Reviews/Wiley**

|  | ((shoulder* OR glenohumeral OR labr*) AND (dislocat* OR luxat* OR subluxat* OR instabilit* OR unstable OR instable OR injur* OR pain)) AND (surger* OR surgic* OR surgeon* OR operation* OR operative* OR invasive* OR restorati* OR reconstruct* OR bony OR Bankart OR SLAP* OR superior-labr* OR (arthroscopic AND stabili*) OR latarjet) AND ((resum* OR return* OR continu* OR discontinu*) AND (sport* OR play* OR activit* OR competit* OR function* OR athlete*)) in Title Abstract Keyword | 37 |
| --- | --- | --- |

**Cochrane Central Register of Controlled Trials/Wiley**

|  | ((shoulder* OR glenohumeral OR labr*) AND (dislocat* OR luxat* OR subluxat* OR instabilit* OR unstable OR instable OR injur* OR pain)) AND (surger* OR surgic* OR surgeon* OR operation* OR operative* OR invasive* OR restorati* OR reconstruct* OR bony OR Bankart OR SLAP* OR superior-labr* OR (arthroscopic AND stabili*) OR latarjet) AND ((resum* OR return* OR continu* OR discontinu*) AND (sport* OR play* OR activit* OR competit* OR function* OR athlete*)) in Title Abstract Keyword | 515 |
| --- | --- | --- |

**SportDiscus/EBSCO**

| # | Query | Results |
| --- | --- | --- |
| S6 | S3 AND S4 AND S5 | 810 |
| S5 | DE "SHOULDER injuries" OR DE "SHOULDER joint injuries" OR (S1 AND S2) | 12,485 |
| S4 | DE "SPORTS participation" OR ( TI ((resum* OR return* OR continu* OR discontinu*) AND (sport* OR play* OR activit* OR competit* OR function* OR athlete* OR DE "ATHLETES")) ) OR ( AB ((resum* OR return* OR continu* OR discontinu*) AND (sport* OR play* OR activit* OR competit* OR function* OR athlete* OR DE "ATHLETES")) ) | 49,403 |
| S3 | DE "SURGERY" OR DE "OPERATIVE surgery" OR DE "SURGICAL procedures" OR (TI (surger* or surgic* or surgeon* or operation* or operative* or invasive* or restorati* or reconstruct* or bony or Bankart or SLAP* OR superior-labr* OR (arthroscopic and stabili*) or Latarjet) OR AB (surger* or surgic* or surgeon* or operation* or operative* or invasive* or restorati* or reconstruct* or bony or Bankart or SLAP* OR superior-labr* OR (arthroscopic and stabili*) or Latarjet)) | 88,443 |
| S2 | DE "JOINT dislocations" OR DE "PAIN" OR ( TI (dislocat* OR luxat* OR subluxat* OR instabilit* OR unstable OR instable OR injur* OR pain) ) OR ( AB (dislocat* OR luxat* OR subluxat* OR instabilit* OR unstable OR instable OR injur* OR pain)) | 171,666 |
| S1 | DE "SHOULDER" OR DE "SHOULDER joint" OR DE "GLENOHUMERAL joint" OR ( TI (shoulder* OR glenohumeral OR labr*) ) OR ( AB (shoulder* OR glenohumeral OR labr*) ) | 26,222 |

**Web of Science, Core collection/Clarivate Analytics**

|  | Topic (((shoulder* OR glenohumeral OR labr*) AND (dislocat* OR luxat* OR subluxat* OR instabilit* OR unstable OR instable OR injur* OR pain)) AND (surger* OR surgic* OR surgeon* OR operation* OR operative* OR invasive* OR restorati* OR reconstruct* OR bony OR Bankart OR SLAP* OR superior-labr* OR (arthroscopic AND stabili*) OR latarjet) AND ((resum* OR return* OR continu* OR discontinu*) AND (sport* OR play* OR activit* OR competit* OR function* OR athlete*))) | 2640 |
| --- | --- | --- |

|  | **October 19th 2021** | | **August 11th 2022 - update** | | **Unique for update** |
| --- | --- | --- | --- | --- | --- |
| **Database** | **Before deduplication** | **After deduplication** | **Before deduplication** | **After deduplication** |  |
| PubMed | 2835 | 2821 | 3069 | 3059 | 226 |
| OVID Embase | 2833 | 1123 | 3143 | 1266 | 196 |
| Cochrane Reviews | 36 | 28 | 37 | 29 | 1 |
| Cochrane CENTRAL | 479 | 282 | 515 | 310 | 31 |
| Web of Science | 2363 | 449 | 2640 | 516 | 71 |
| SportDiscus | 764 | 131 | 810 | 140 | 14 |
| **Total** | 9310 | 4834 | 10214 | 5320 | 539 |
