# Supplementary material for: Why do patients with anterior shoulder instability not return to sport after surgery? A systematic review of 63 studies comprising 3545 patients
Source: JSES Int. 2023 Jan 20;7(3):376–84. doi: 10.1016/j.jseint.2023.01.001 (PMC10229421; doi:10.1016/j.jseint.2023.01.001)
Supplement: Supplementary Appendix S2 [file mmc2.docx]

| **First Author; Journal** | **Year** | **Surgery Type** | **Patients; Shoulders, n** | **Level of Athletics, n** | **Mean ± SD Age at Surgery (Range), y** | **Male:**  **Female, n** | **Mean ± SD Follow-up (Range), mo** | **MINORS Score/RoB-2 Score** |
| --- | --- | --- | --- | --- | --- | --- | --- | --- |
| Alentorn-Geli; *Knee Surg Sports Traumatol Arthrosc^b^* | 2015 | Arthroscopic Bankart Repair | 57; 57 | 57 competitive/professional | Median 22.0 (16.0-28.0) | 57; 0 | Median 96.0 (60.0-120.0) | 9 |
| Arianjam; *Shoulder Elbow^b^* | 2015 | Open Latarjet Procedure combined with Arthroscopic Bankart Repair | 34; 34 | NA*^a^* | 21.0 (15.0-29.0) | 34; 0 | 36.0 (24.0-60.0) | 10 |
| Bacilla; *Artrosc J Arthrosc Relat Surg^c^* | 1997 | Arthroscopic Bankart Repair | 40; 40 | 8 recreational; 32 competitive/professional | 18.0 (16.0-27.0) | NA*^a^* | 30.0 (18.0-36.0) | 7 |
| Belangero; *J Shoulder Elbow Surg^d^* | 2021 | Open Bristow Procedure &  Open Latarjet Procedure | 37; 41 | 26 recreational; 15 competitive/professional | 26.4 (16.0-46.0) | 37; 4 | 60.0 **±** NA*^a^* | Low Risk |
| Blonna; *Am J Sports Med^b^* | 2013 | Arthroscopic Bankart Repair &  Open Latarjet Procedure | 60; 60 | NA*^a^* | 31.5 (19.0-53.0) | 52; 8 | 70.8 (24.0-108.0) | 17 |
| Bohu; *Orthop Traumatol Surg^c^* | 2016 | Open Latarjet Procedure | 46; 46 | 4 none, 8 recreational;  34 competitive/professional | 25.3 **±** 6.4 | 41; 5 | 18.5 ± 5.2 | 21 |
| Brandariz; *Artrosc J Arthrosc Relat Surg^b^* | 2015 | Open Latarjet Procedure | 29; 29 | 7 recreational; 22 competitive/professional | 30.0 **±** 2.0 | 27; 2 | 35.0 ± 6.0 | 11 |
| Cordasco; *J Shoulder Elbow Surg^c^* | 2020 | Arthroscopic Bankart Repair | 67; 67 | 67 competitive/professional | 17.5 (13.0-21.0) | 48; 19 | 42.7 (24.0-96.0) | 11 |
| Cruz; *J Shoulder Elbow Surg^b^* | 2021 | Open Latarjet Procedure | 46; 46 | 46 competitive/professional | 23.1 (18.0-31.0) | NA*^a^* | 37.2 (24.0-78.0) | 9 |
| Castrini; *Artrosc J Arthrosc Relat Surg^b^* | 2022 | Arthroscopic Latarjet Procedure | 93; 95 | NA*^a^* | 28.7 **±** 8.1 (18.0-52.0) | 88; 5 | 69.5 ± 23.8 (36.0-110.0) | 10 |
| De Carli; *Int Orthop^b^* | 2019 | Open Capsulopasty &  Open Latarjet Procedure | 73; 73 | NA*^a^* | 28.0 (16.0-41.0) | 48; 25 | 72.0 (72.0-108.0) | 16 |
| Davey; *Artrosc Sports Med and Rehab^b^* | 2021 | Arthroscopic Bankart Repair | 200; 200 | NA*^a^* | 27.2 **±** 8.0 | 174; 26 | 62.0 **±** 20.6 | 17 |
| Dekker; *Orthop J Sports Med^c^* | 2020 | Arthroscopic Bankart Repair &  Open Latarjet Procedure | 23; 25 | 25 competitive/professional | 24.3 **±** 4.9 (16-35) | 20; 3 | NA*^a^* | 18 |
| Dickens; *Am J Sports Med^c^* | 2017 | Arthroscopic Bankart Repair | 29; 29 | 29 competitive/professional | NA*^a^* | NA*^a^* | NA*^a^* | 17 |
| Domos; *Shoulder Elbow^b^* | 2019 | Arthroscopic Bankart Repair with/without remplissage | 40; 40 | 40 competitive/professional | 25.0 (15.0-40.0) | 38; 2 | 26.0 (24.0-27.0) | 9 |
| **First Author; Journal** | **Year** | **Surgery**  **Type** | **Patients; Shoulders, n** | **Level of Athletics, n** | **Mean ± SD Age at Surgery (Range), y** | **Male:**  **Female, n** | **Mean ± SD Follow-up (Range), mo** | **MINORS Score/RoB-2 Score** |
| Ee; *J Orthop Surg Res^b^* | 2011 | Arthroscopic Bankart Repair | 75; 79 | NA*^a^* | 24.9 (13.0-44.0) | 174; 1 | 24.0 **±** NA*^a^* |  |
| Fabre; *J Shoulder Elbow Surg^b^* | 2010 | Open Bankart Repair | 49; 50 | 3 none, 7 recreational; 39 competitive/professional | 25.0 (17.0-51.0) | 46; 3 | 28.0 (25.0-32.0) | 10 |
| Fremery; *Int J Sports Med^c^* | 2006 | Arthroscopic Bankart Repair | 22; 20 | NA*^a^* | 22.2 ± 2.6 (18.0-30.0) | 15; 7 | 33.6 ± 8.4 | 14 |
| Gartsman; *J Bone Joint Surg Am^c^* | 2000 | Arthroscopic Bankart Repair | 53; 53 | 10 none, 35 recreational; 8 competitive/professional | 32.0 (15.0-58.0) | 44; 9 | 33.0 (36.0-63.0) | 13 |
| Gerometta; *Knee Surg Sports Traumatol Arthrosc^c^* | 2016 | Arthroscopic Bankart Repair | 46; 46 | 30 recreational; 16 competitive/professional | 28.9 ± 8.1 (12.0-35.0) | 37; 9 | 24.4 ± 7.7 | 9 |
| Goldberg; *Am J Sports Med^b^* | 2016 | Arthroscopic Capsulolabral Repair | 37; 38 | 2 none, 15 recreational; 20 competitive/professional | 25.0 (13.0-44.0) | 25; 12 | 36.0 (24.0-54.0) | 8 |
| Harada; J *Orthop Sci^b^* | 2021 | Arthroscopic Capsulolabral Repair | 24; 24 | 24 competitive/professional | 17.6 ± NA*^a^* | 13; 11 | 39.7 ± NA*^a^* | 10 |
| Hayashida; *Artrosc J Arthrosc Relat Surg^b^* | 2006 | Arthroscopic Bankart Repair | 47; 47 | 47 competitive/professional | 26.0 (16.0-49.0) | 38; 9 | 28.0 (24.0-38.0) | 9 |
| Hurley; *Surg-J R Coll Surg E^b^* | 2021 | Arthroscopic Bankart Repair | 208; 208 | NA*^a^* | 28.6 ± NA*^a^* | 184; 24 | 62.7 ± NA*^a^* | 14 |
| Hurley; *Orth J Sports Med^b^* | 2022 | Open Latarjet Procedure | 105; 105 | NA*^a^* | 26.8 ± 6.0 | 105; 0 | 40.0 ± 25.0 | 16 |
| Ide; *Am J Sports Med^c^* | 2004 | Arthroscopic Bankart Repair | 55; 55 | 10 recreational; 45 competitive/professional | 19.5 (15.0-39.0) | 41; 14 | 42.0 (25.0-72.0) | 14 |
| Iizawa; *Knee Surg Sports Traumatol Arthrosc^b^* | 2020 | Arthroscopic Bankart Repair & Open Latarjet Procedure | 68; 68 | NA*^a^* | 25.2 (13.0-72.0) | 57; 11 | 31.1 (24.0-79.0) | 18 |
| Jobe; *Am J Sports Med^b^* | 1991 | Open Capsulolabral Repair | 25; 25 | 20 competitive/professional | 21.0 (15.0-27.0) | 25; 0 | 39.0 (27.0-53.0) | 6 |
| John; *Artrosc J Arthrosc Relat Surg^b^* | 2007 | Arthroscopic Capsulolabral Repair | 33; 33 | NA*^a^* | 25.2 (15.0-54.0) | 23; 13 | 35.0 (24.0-41.0) | 7 |
| Jones; *Am J Sports Med^b^* | 2012 | Arthroscopic Capsular Plication | 20; 20 | 20 competitive/professional | 19.1 (12.0-26.0) | 7; 13 | 43.2 (24.0-66.0) | 10 |
| Kjeldsen; *Scand J Med Sci Sports^c^* | 1996 | Open Bankart Repair | 16; 16 | 3 recreational; 13 competitive/professional | 28.4 (17.0-47.0) | 13; 3 | 23.9 (16.0-36.0) | 13 |
| **First Author; Journal** | **Year** | **Surgery Type** | **Patients; Shoulders, n** | **Level of Athletics, n** | **Mean ± SD Age at Surgery (Range), y** | **Male:**  **Female, n** | **Mean ± SD Follow-up (Range), mo** | **MINORS Score/RoB-2 Score** |
| Levine; *Am J Sports Med^b^* | 2000 | Arthroscopic Capsular Shift | 50; 50 | NA*^a^* | 27.0 (27.0-49.0) | 33; 17 | 56.4 (24.0-115.0) | 8 |
| Levy; *Artrosc J Arthrosc Relat Surg^b^* | 2007 | Open Bankart Repair | 36; 37 | NA*^a^* | 25.0 (16.0-49.0) | 35; 1 | 36.0 (27.0-87.0) | 8 |
| Levy; *Am J Orthop^b^* | 2014 | Arthroscopic Capsulolabral Repair | 20; 20 | NA*^a^* | 20.2 ± 7.3 (15.0-36.4) | 15; 5 | 40.8 ± 7.2 (32.4-61.2) | 8 |
| Lima; *J Shoulder Elbow Surg Int^b^* | 2022 | Open Latarjet Procedure | 13; 13 | 13 competitive | 29.2 ± 9.5 (15.0-46.0) | 0; 13 | NA*^a^* | 9 |
| Lunn; *J Shoulder Elbow Surg^b^* | 2008 | Open Eden-Hybinette Procedure | 34; 34 | NA*^a^* | 30.0 (18.4-51.8) | 27; 7 | 81.7 (24.0-206.4) | 9 |
| Marcacci; *Artrosc J Arthrosc Relat Surg^b^* | 1996 | Arthroscopic Capsulolabral Repair | 71; 71 | 4 none, 44 recreational; 23 competitive/professional | 25.4 (16.0-51.0) | 60; 11 | 46.6 (26.0-75.0) | 17 |
| Marquardt; *Am J Orthop^b^* | 2005 | Arthroscopic Capsulolabral Repair | 35; 38 | 9 none, 23 recreational; 6 competitive/professional | 25.4 (15.0-55.0) | 9; 26 | 88.8 (48.0-136.8) | 12 |
| Massoud; *J Shoulder Elbow Surg^b^* | 2002 | Arthroscopic Bankart Repair | 59; 59 | 15 none, 41 recreational; 3 competitive/professional | 27.0 (16.0-53.0) | 52; 7 | 42.0 (24.0-58.0) | 7 |
| McLeod; *Ir J Med Sci^b^* | 2021 | Arthroscopic Bankart Repair | 54; 57 | 37 recreational; 17 competitive/professional | 22.6 (15.0-43.0) | 47; 7 | 24.0 (7.0-48.0) | 8 |
| Milchteim; *Artrosc J Arthrosc Relat Surg^b^* | 2015 | Arthroscopic Bankart Repair | 89; 94 | 8 recreational; 86 competitive/professional | 21.9 (15.0-43.0) | 87; 2 | 60.0 (36.0-105.6) | 11 |
| Mishra; *Indian J Orthop^b^* | 2012 | Arthroscopic Bankart Repair | 50; 50 | 40 recreational; 10 competitive/professional | 26.8 (18.0-45.0) | NA*^a^* | 27.0 (24.0-36.0) | 9 |
| Neviaser; *J Shoulder Elbow Surg^b^* | 2017 | Open Bankart Repair | 127; 127 | NA*^a^* | 31.0 (15.0-63.0) | 102; 25 | 205.2 (60.0-288.0) | 11 |
| Neyton; *J Shoulder Elbow Surg^b^* | 2012 | Open Latarjet Procedure | 34; 37 | 34 competitive/professional | 23.4 (15.0-30.0) | 34; 0 | 144.0 ± NA*^a^* | 10 |
| Ortmaier; *Orthop Traumatol Surg Res^b^* | 2019 | Open Iliac Crest Bone Graft Augmentation | 34; 34 | 27 recreational; 7 competitive/professional | 26.9 ± 12.3 | 29; 5 | 39.5 ± 9.6 (25.0-56.0) | 17 |
| Owens; *Am J Sports Med^b^* | 2009 | Arthroscopic Bankart Repair | 39; 40 | 39 competitive/professional | 20.3 (17.0-23.0) | 37; 2 | 140.4 (109.2-166.8) | 9 |
| Pagnani; *J Bone Joint Surg Am^b^* | 2002 | Open Bankart Repair | 58; 58 | 58 competitive/professional | 18.2 (15.0-29.0) | 58; 0 | 37.0 ± NA*^a^* | 7 |

| **First Author; Journal** | **Year** | **Surgery Type** | **Patients; Shoulders, n** | **Level of Athletics, n** | **Mean ± SD Age at Surgery (Range), y** | **Male:**  **Female, n** | **Mean ± SD Follow-up (Range), mo** | **MINORS Score/RoB-2 Score** |
| --- | --- | --- | --- | --- | --- | --- | --- | --- |
| Park; *Artrosc J Arthrosc Relat Surg^b^* | 2018 | Arthroscopic Bankart Repair | 32; 32 | NA*^a^* | 21.9 (15.0-43.0) | 29; 3 | 39.4 ± 14.3 | 19 |
| Plath; *Artrosc J Arthrosc Relat Surg^b^* | 2015 | Arthroscopic Bony Bankart Repair | 45; 45 | 1 none, 41 recreational; 3 competitive/professional | 41.2 ± 15.4 (15.0-71.0) | 36; 9 | 82.0 ± 31.0 | 11 |
| Privitera; *Am J Sports Med^b^* | 2012 | Arthroscopic Bankart Repair | 20; 20 | NA*^a^* | 43.0 (28.0-73.0) | 20; 0 | 162.0 (129.0-210.0) | 11 |
| Ranalleta; *Orthop J Sports Med^b^* | 2017 | Arthroscopic Bankart Repair | 20; 20 | 20 competitive/professional | 25.4 (18.0-35.0) | 16; 4 | 71.0 (36.0-96.0) | 12 |
| Ranalleta; *Am J Sports Med^b^* | 2018 | Arthroscopic and Open Bankart Repair | 48; 49 | 49 competitive/professional | 22.8 (17.0-35.0) | 48; 0 | 48.0 (24.0-108.0) | 12 |
| Roberts; *J Shoulder Elbow Surg^b^* | 1999 | Arthroscopic and Open Capsulolabral Repair | 52; 56 | 52 competitive/professional | NA*^a^* | 52; 0 | 29.4 (12.0-65.0) | 8 |
| Rossi; *Am J Sports Med^b^* | 2020 | Open Latarjet Procedure | 145; 145 | 145 competitive/professional | 25.3 (18.0-45.0) | 135; 10 | 41.3.0 (24.0-90.0) | 19 |
| Rossi; *Orthop J Sports Med^b^* | 2021 | Arthroscopic Bankart Repair | 208; 208 | 99 recreational, 109 competitive/professional | 24.0 (18.0-30.0) | 176; 32 | 44.0 (24.0-90.0) | 12 |
| Saper; *Orthop J Sports Med^b^* | 2017 | Arthroscopic Bankart Repair | 37; 39 | 39 competitive/professional | 16.9 ± 1.5 | 38; 1 | 75.2 ± 18.5 | 10 |
| Saper; *Clin J Sport Med^b^* | 2021 | Arthroscopic Bankart Repair | 33; 33 | 33 competitive/professional | 23.8 (18.0-33.0) | 33; 0 | 75.6 (49.2-111.6) | 10 |
| Tjong; *Am J Sports Med^b^* | 2015 | Arthroscopic Bankart Repair | 25; 25 | 15 recreational; 10 competitive/professional | 27.6 (18.0-40.0) | 24; 1 | 24.0 ± NA*^a^* | 8 |
| Uchiyama; *Am J Sports Med^b^* | 2009 | Open Capsular Shift | 50; 52 | 50 competitive/professional | 20.4 (14.0-38.0) | 42; 8 | 60.8 (24.0-172.0) | 7 |
| Venkatachalam; *Shoulder Elbow^b^* | 2016 | Open Sheffield Bone Block Procedure | 84; 84 | NA*^a^* | 33.0 (16.0-45.0) | 59; 25 | 48.0 (36.0-84.0) | 9 |
| Yamamoto; *Orthop Traumatol Surg Res^b^* | 2015 | Arthroscopic and Open Bankart Repair | 100; 100 | NA*^a^* | 24.0 (14.0-54.0) | 76; 24 | 17.0 (12.0-96.0) | 16 |
| Yee; *Aust NZ J Surg^b^* | 1999 | Arthroscopic Capsulolabral Repair & Open Bony Procedure | 37; 38 | 21 recreational; 16 competitive/professional | 24.0 (17.0-44.0) | 34; 3 | 55.2 (12.0-90.0) | 7 |

Supplement 2 Data for Included Surgical Treatment of Shoulder Instability Studies^a^

*^a^*NA, Not Available *^c^*Level of evidence: 2

*^b^*Level of evidence: 3 ^d^Level of evidence: 1
